# Complete sequences of two *Paenibacillus* sp. strains and one *Lysinibacillus* strain isolated from the environment on STAA medium highlight biotechnological potential

**DOI:** 10.1128/mra.00199-24

**Published:** 2024-04-29

**Authors:** Sabrina A. Attéré, Laurie C. Piché, Laurent Intertaglia, Raphaël Lami, Steve J. Charette, Antony T. Vincent

**Affiliations:** 1 Institut de Biologie Intégrative et des Systèmes, Pavillon Charles-Eugène-Marchand, Université Laval, Quebec City, Quebec, Canada; 2 Centre de Recherche de l'Institut Universitaire de Cardiologie et de Pneumologie de Québec, Hôpital Laval, Quebec City, Quebec, Canada; 3 Département de biochimie, de microbiologie et de bio-informatique, Faculté des Sciences et de Génie, Université Laval, Quebec City, Quebec, Canada; 4 Département des sciences animales, Faculté des Sciences de l'Agriculture et de l'Alimentation, rue de l'Agriculture, Université Laval, Quebec City, Quebec, Canada; 5 Sorbonne Université, CNRS, Bio2mar, Observatoire Océanologique de Banyuls-sur-Mer, Avenue Pierre Fabre, Banyuls-sur-Mer, France; 6 Sorbonne Université, CNRS, Laboratoire de Biodiversité et Biotechnologies Microbiennes, Observatoire Océanologique de Banyuls-sur-Mer, Avenue Pierre Fabre, Banyuls-sur-Mer, France; Indiana University, Bloomington, Bloomington, Indiana, USA

**Keywords:** biotechnology, environmental microbiology, *Lysinibacillus*, *Paenibacillus*, secondary metabolites, STAA medium

## Abstract

Streptomycin thallous acetate actidione medium is typically used to isolate *Brochothrix thermosphacta* bacteria from food. Using this medium, three bacterial strains were isolated from the environment. Genomic sequences demonstrated that these bacteria are of the genera *Lysinibacillus* and *Paenibacillus* and are of biotechnological interest.

## ANNOUNCEMENT

Streptomycin thallous acetate actidione (STAA) agar is used to isolate *Brochothrix thermosphacta* bacteria (1), a meat spoilage agent (2). This medium has also been used to isolate soil bacteria, leading to the identification of *Brochothrix campestris* (3), the only other species in the *Brochothrix* genus apart from *thermosphacta*.

A sampling campaign was carried out in autumn of 2023 in Quebec City (Province of Quebec, Canada). Two 7.5-mL soil samples from different sites were each combined with 7.5 mL of water and left at room temperature for 6 h. An aliquot of each sample (1 mL) was spread on STAA agar medium and incubated at 25°C for 48 h. A water sample from a watering hole located in a wooded area was also plated on STAA agar medium and underwent incubation at 25°C for 48 h. White colonies were replated onto HIB agar and incubated at 25°C for 48 h. One isolate per sample (i.e., Y5S-7 [soil], Y5S-8 [soil], and Y5S-9 [water]), was frozen at −80°C in HIB liquid medium with 20% glycerol as a cryoprotectant.

Strains Y5S-7, Y5S-8, and Y5S-9 were thawed on HIB agar medium and incubated for 24–48 h at 25°C. The total DNA of the strains was extracted with QIAamp PowerFecal Pro DNA Kit (QIAGEN, Toronto, ON, Canada) following the manufacturer’s instructions. DNA samples were sequenced by Plasmidsaurus (Eugene, OR, USA) using an Illumina NextSeq2000 (2 × 150 bp) and a Nanopore PromethION. The Illumina libraries were prepared using the SeqWell ExpressPlex 96 library prep kit, while the Nanopore libraries were prepared using v14 library prep chemistry without fragmentation or size selection, and sequenced on an R10.4.1 flow cell. Basecalling was carried out for Nanopore reads using Dorado version 7.1.4 (4) on super-accurate mode. Illumina reads were filtered using Fastp version 0.23.4 (5) with default parameters, while Nanopore reads were filtered using Filtlong version 0.2.1 (6) and retained the best 90% of reads above 1,000 bp or until only 500 Mbp remained. After filtration, 13.28 million, 5.84 million, and 9.03 million Illumina reads remained for Y5S-7, Y5S-8, and Y5S-9, respectively. A total of 86,759 (N50 = 6,329 bp), 78,181 (N50 = 6,804 bp), and 98,146 (N50 = 6,131 bp) Nanopore reads were retained for strains Y5S-7, Y5S-8, and Y5S-9, respectively. Read quality was verified with FastQC version 0.12.1 (7). A hybrid assembly was then performed for each data set using Unicycler version 0.5.0 (8). The sequences were linearized to the *dnaA* gene for chromosomes and *repA* for plasmids using Unicycler version 0.5.0 (8), and were annotated by the NCBI using PGAP (9) on submission to GenBank. The assembly characteristics are shown in Table 1.

**TABLE 1:** Genomic characteristics of Y5S-7, Y5S-8, and Y5S-9 strains

Genus	Species	Strain	Replicon	Length (bp)	CDSs	GC (%)	Coverage (×)	GenBank
*Lysinibacillus*	sp.	Y5S-8	Chromosome	4,672,069	4,442	37.78	194	CP145889
			pY5S8-1	4,614	6	41.46	46	CP145890
			pY5S8-2	2,217	2	41.5	80	CP145891
*Paenibacillus*	sp.	Y5S-9	Chromosome	6,924,530	5,927	45.94	195	CP145887
			pY5S9-1	4,690	5	44.43	63	CP145888
*Paenibacillus*	*amylolyticus*	Y5S-7	Chromosome	6,917,693	5,933	45.97	268	CP145892
			pY5S7-1	374,980	272	38.55	354	CP145893
			pY5S7-2	68,778	83	39.56	1,079	CP145894

Taxonomic identification using the Type Strain Genome Server (10) suggests that Y5S-8 and Y5S-9 may be novel species within the *Lysinibacillus* and *Paenibacillus* genera, respectively, while Y5S-7 was identified as a *Paenibacillus amylolyticus* species. Annotation with antiSMASH (11) demonstrated that strains Y5S-7 and Y5S-9 have all the genes to produce bacillopaline, a metallophore (12), and polymyxin, a last-resort antibiotic (13) with biotechnological potential.

## Data Availability

The chromosomal and plasmid sequences of Y5S-7, Y5S-8, and Y5S-9 strains have been deposited in GenBank (Table 1). Illumina and Nanopore sequencing reads were deposited in the Sequence Read Archive (SRA) database: Y5S-7 (SRX23698269 and SRX23698272), Y5S-8 (SRX23698270 and SRX23698273), and Y5S-9 (SRX23698271 and SRX23698274).
